# CT-based pancreatic radiomics predicts secondary loss of response to infliximab in biologically naïve patients with Crohn’s disease

**DOI:** 10.1186/s13244-024-01637-4

**Published:** 2024-03-13

**Authors:** Tian Yang, Jing Feng, Ruchen Yao, Qi Feng, Jun Shen

**Affiliations:** 1 Renji Hospital, School of Medicine, Shanghai Jiao Tong University; Division of Gastroenterology and Hepatology, Key Laboratory of Gastroenterology and Hepatology, Ministry of Health, Inflammatory Bowel Disease Research Center, Shanghai Institute of Digestive Disease, 160# Pu Jian Ave, Shanghai, 200127 China; 2 NHC Key Laboratory of Digestive Diseases (Renji Hospital, Shanghai Jiaotong University School of Medicine), Shanghai, China; 3 Department of Radiology, Renji Hospital, School of Medicine, Shanghai Jiao Tong University, 160 Pu Jian Road, Shanghai, 200127 China

**Keywords:** Crohn’s disease, Pancreas, Radiomics, Secondary loss of response, Computed tomography enterography

## Abstract

**Objectives:**

Predicting secondary loss of response (SLR) to infliximab (IFX) is paramount for tailoring personalized management regimens. Concurrent pancreatic manifestations in patients with Crohn’s disease (CD) may correlate with SLR to anti-tumor necrosis factor treatment. This work aimed to evaluate the potential of pancreatic radiomics to predict SLR to IFX in biologic-naive individuals with CD.

**Methods:**

Three models were developed by logistic regression analyses to identify high-risk subgroup prone to SLR. The area under the curve (AUC), calibration curve, decision curve analysis (DCA), and integrated discrimination improvement (IDI) were applied for the verification of model performance. A quantitative nomogram was proposed based on the optimal prediction model, and its reliability was substantiated by 10-fold cross-validation.

**Results:**

In total, 184 CD patients were enrolled in the period January 2016 to February 2022. The clinical model incorporated age of onset, disease duration, disease location, and disease behavior, whereas the radiomics model consisted of five texture features. These clinical parameters and the radiomics score calculated by selected texture features were applied to build the combined model. Compared to other two models, combined model achieved favorable, significantly improved discrimination power (AUC_combined vs clinical_ 0.851 vs 0.694, *p* = 0.02; AUC_combined vs radiomics_ 0.851 vs 0.740, *p* = 0.04) and superior clinical usefulness, which was further converted into reliable nomogram with an accuracy of 0.860 and AUC of 0.872.

**Conclusions:**

The first proposed pancreatic-related nomogram represents a credible, noninvasive predictive instrument to assist clinicians in accurately identifying SLR and non-SLR in CD patients.

**Critical relevance statement:**

This study first built a visual nomogram incorporating pancreatic texture features and clinical factors, which could facilitate clinicians to make personalized treatment decisions and optimize cost-effectiveness ratio for patients with CD.

**Key points:**

• The first proposed pancreatic-related model predicts secondary loss of response for infliximab in Crohn’s disease.

• The model achieved satisfactory predictive accuracy, calibration ability, and clinical value.

• The model-based nomogram has the potential to identify long-term failure in advance and tailor personalized management regimens.

**Graphical Abstract:**

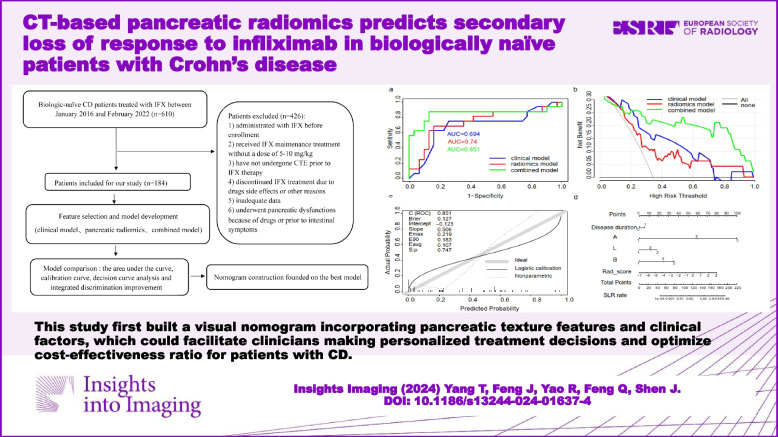

**Supplementary Information:**

The online version contains supplementary material available at 10.1186/s13244-024-01637-4.

## Introduction

Crohn’s disease (CD) is a chronic and relapsing intestinal disorder with numerous extraintestinal manifestations (EIMs) including dysfunctions in the eyes, joints, skin, and pancreas [1]. The increasing global incidence of this disease is bringing a heavy healthcare burden, predominantly caused by drugs like anti-tumor necrosis factor (anti-TNF) biologics [2, 3]. Anti-TNF antibodies have been recognized as a revolutionary therapy for CD, facilitating mucosal healing, reducing the need for surgeries and hospitalizations, and raising patient’s quality of life [4, 5]. However, studies have concluded that up to half of individuals demonstrate secondary loss of response (SLR) to infliximab (IFX) [6]. In addition, some patients may experience unpredictable fatal side effects due to individual variation in drug response. Given the advent of selective, novel biological agents, such as JAK and α4β7 integrin inhibitors, the identification of SLR to IFX in advance is essential for determining appropriate treatment protocols and optimizing the cost-effectiveness ratio.

Multiple studies have verified that patient-related factors, disease characteristics, and serological indicators can predict the occurrence of SLR to IFX in CD. These factors include body mass index [7], non-stricturing and non-penetrating disease [8], and C-reactive protein (CRP) [9]. However, only scant studies have explored the latent association between coexisting conditions and treatment failure in patients with CD. Accumulated research has revealed pancreatic involvement in individuals with CD, including acute and chronic pancreatitis, pancreatic exocrine insufficiency, autoimmune pancreatitis (AIP), asymptomatic pancreatic enzyme elevations, and silent imaging abnormalities [10–12]. The heterogeneous group of pancreatic conditions is recognized as EIMs of inflammatory bowel disease (IBD) or IBD therapeutic consequence, but can also exist as a comorbidity [13]. To date, the linkage between pancreatic entities and IBD activity remains controversial given the inconclusive data. Individuals with active CD seem to confer risk for pancreatitis and AIP [14–16]. A cross-sectional work has suggested that elevated levels of pancreatic enzymes in IBD population hold relevance to a more active disease phenotype and mucosal histological activity [17]. Nevertheless, other research has not discovered the degree of enzymes elevation and activity of CD is correlated [18]. Several studies regarding autoimmune diseases have demonstrated that individuals with comorbidities have a lower likelihood to benefit from biologics than individuals without [19, 20]. Considering the present evidence, it is plausible that pancreatic diseases in individuals with CD might play an essential role in predicting treatment failure to IFX. Indeed, research has shown that hepato-pancreato-biliary conditions are closely correlated with the primary loss of response to anti-TNF treatments in patients with IBD [21].

Texture analysis (TA) is an image analysis technique that extracts and quantifies subtle lesion features by analyzing the gray-scale values of regions of interest (ROI), especially in substantive organs, serving as a promising predictive tool for postoperative complications, therapeutic outcomes, disease prognosis, and other applications [22–24]. Computed tomography enterography (CTE) is widely used to monitor inflammatory lesions and make a transluminal diagnosis of CD in clinic [25]. Based on recently published research, a subgroup of CD individuals with a higher probability of SLR has been identified by TA of intestinal lesions in CTE images [26]. Furthermore, the indicators of substantive organs are more stable compared to hollow organs. Thus, our work attempted to elucidate the underlying predictive values of CTE pancreatic texture parameters for identifying SLR to IFX in patients with CD and to compare the performance of the combined model and the single feature model to discriminate between SLR and non-SLR. Ultimately, a nomogram with visualization based on the most valuable model was formulated, which may provide a convenient means to guide personalized treatment options for individuals with CD.

## Methods

### Patient selection

**Fig. 1:**
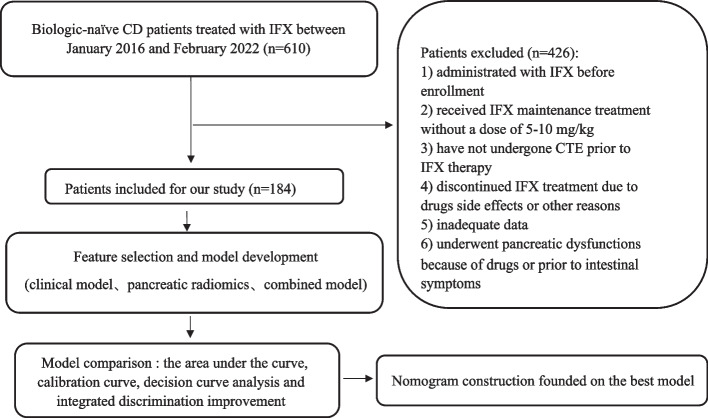
The flowchart of patient recruitment

This work was permitted by the Ethics Committee of Renji Hospital and the demand for obtaining informed consent was exempted given the retrospective property of this research. We retrospectively analyzed the medical data of individuals with CD who had received IFX treatment between January 2016 and February 2022 in our Hospital. Diagnosis of CD was made depending upon the guidelines of the European Crohn’s and Colitis Organization [27]. We recruited patients who were managed with IFX with a dosage of 5–10 mg/kg every 8 weeks during maintenance treatment. Exclusion criteria were as follows: patients with prior administration of IFX; untreated with a dose of 5–10 mg/kg during the induction and/or maintenance period; patients who did not receive CTE within 2 weeks ahead of IFX treatment; and patients who discontinued IFX therapy, had inadequate data, and had pancreatic dysfunctions because of drugs or prior to intestinal symptoms. Clinical baseline characteristics and CTE images were obtained from the medical record database and the imaging system at our institution. A detailed patient enrollment process is illustrated in Fig. 1.

### Outcomes and definitions

At present, a consensus has not been reached on what SLR means, but the recurrence of clinical symptoms after an excellent induction response is considered a rational criterion [28]. In this study, SLR was defined by a multidisciplinary specialists group as a loss of responsiveness during week 14 to week 54 after therapy initiation, involving total Harvey Bradshaw indices (HBI) > 5 or an increase in the HBI ≥ 3 points, dose escalation, interval shortening, need for immunosuppressive therapy, corticosteroids or surgery related to CD, switching to other biological agents, and/or mucosal recurrence (a reduction from baseline in simple endoscopic score for CD (SES-CD) < 50% or SES-CD ≥ 3).

### CTE image acquisition and analysis

CTE examinations were conducted within 2 weeks prior to IFX treatment following the standard protocol. The patients fasted before examination and respectively administered with 500 mL of polyethylene glycol solution (Wanghe Pharma, Shenzhen, China) at 45 min, 30 min, and 15 min prior to the scan. CT scans were performed from the diaphragm to the perineum and completed in a period of a single breath hold adopting two 64-detector CT scanners adopting two 64-detector CT scanners (GE DISCOVERY CT750HD, Milwaukee, WI, USA; UNITED IMAGING, uCT760, Shanghai, China), when patients were in a supine position. Contrast-enhanced scanning was conducted after the intravenous injection of contrast agent (Lopamiro 370, Bracco Sine, Shanghai, China; 1.5 mL/kg) through the dorsal hand vein at the rate of 3 mL/s. The scanning parameters of the GE scanner were as detailed below: tube current, 228 mAs; voltage, 120 kV; collimation, 40 mm; pitch, 1.375:1. The parameters of the UI scanner were as follows: tube current, 206 mAs; voltage, 120 kV; collimation, 80 mm; pitch, 0.994:1. The reconstruction thickness was 1.0 mm. CT images of the enteric phase were attained 70 s after injection of the intravenous contrast agent and retrieved for extracting texture feature.

To measure the pancreas, three ROIs of 100 mm^2^ were placed in the head, body, and tail of the pancreas by two experienced radiologists, surrounded by pancreatic tissue not only in the imaging plane, but also in the adjacent upper and lower planes to minimize the interference of extrapancreatic fat tissue on the volume average [29–33]. In addition, obvious pancreatic lesions, blood vessels, and bile ducts were avoided as much as possible to prevent interference with the texture analysis of pancreatic parenchyma. The head of the pancreas was referred to as the pancreatic region to the right side of the left border of the superior mesenteric vein. The body was referred to the pancreatic parenchyma between the left edge of the superior mesenteric vein and the left edge of the aorta, and the tail was referred to as the remanent pancreatic areas between the left border of the aorta and the splenic hilum [34]. The mean of all three ROIs in the pancreas determined the overall pancreatic parameters [31–33]. The consistency and reproducibility of judgement between different researchers were estimated using two-way random single measures intraclass correlation coefficient (ICC) on 30 randomly selected CT image sets, which achieved a value of 0.876 (*p* < 0.001).

CTE images with the DICOM format were directly imported into Local Image Features Extraction (LIFEx) version 5.10 software (http://www.lifexsoft.org) to implement TA analysis (Image Biomarker Standardization Initiative standard-compliant) [35, 36]. Before feature extraction, we applied voxel resampling and gray-levels absolute discretization to increase consistency between images. Images were resampled to an isotropic 1 mm^3^ voxel size. Intensity discretization was performed in absolute scale bounds between -1000 and 3000 HU with 400 bins (bin-width 10 HU). Then, two well-experienced radiologists used 2D partitioning technique to manually delineate ROIs, which were automatically processed by the software to compute pancreatic features [37]. A total of 44 radiomics features were obtained, consisting of conventional and histogram-founded parameters and second- and higher-order texture indices, as listed in detail in Additional file 1 (supplementary material).

### Model construction

To establish the clinical model, univariate analysis was performed to screen potential variables. Those with a *p* < 0.2 were utilized for further screening [38]. Backward stepwise multivariate logistic regression was conducted to determine the best combination of clinical variables with the minimal Akaike information criterion (AIC).

To construct the radiomics model, the *z*-score method, suitable for normalizing radiomics parameters with different magnitude orders, was adopted to improve the homogeneity of all radiomics features [39], among which variables with ICC > 0.8 were used to screen further. The least absolute shrinkage and selection operator (LASSO) with 10-fold cross-validation was conducted for determining the subset of features with the best λ, and then building the radiomics model by backward stepwise multivariate logistic regression.

In order to comprehensively consider clinical and radiomics features, the radiomics score (rad_score) was formulated with the selected texture features weighted by their respective coefficients. Finally, the combined model incorporating clinical factors and the rad_score was built by logistic regression.

### Model comparison and nomogram development

Comparison of models was mainly based on the validation set. The receiver operating characteristic (ROC) curve and calibration curve was generated to estimate the discriminatory performance and prediction consistence. The area under the ROC curve (AUC) and AIC values of the different models were also calculated. Integrated discrimination improvement (IDI) and Decision curve analysis (DCA) was performed to reflect the improvement and clinical significance of models. Rest on the optimal model, and user-friendly nomogram were formulated for differentiating patients with SLR from those without it (non-SLR), and its reliability was corroborated by 10-fold cross-validation.

### Statistical analysis

The data analysis was conducted by Stata 15.0 and R version 4.0.5. Categorical variables are denoted by number or percentage, and continuous variables are denoted by medians (interquartile ranges). The Fisher’s exact test and Wilcoxon rank-sum tests were respectively applied for categorical and continuous variable comparisons. Reliability analysis was performed using the ICC with a two-way random model. Discrepancies in the AUC values between models were evaluated using DeLong analysis. A two-sided *p* value less than 0.05 was regarded as statistically different.

## Results

### Baseline characteristics

A total of 184 biologic-naïve CD patients treated with IFX were recruited, and subsequently were randomly allocated into training set and validation set, among which 54 (39.4%) and 16 (34.0%) patients did not get sufficient remission from IFX, respectively. There were no statistical differences between two datasets in any of the presented clinical characteristics in Table 1 (all *p* > 0.05).

**Table 1:** Baseline characteristics of CD patients

Characteristics	Training	Validation	*p* value
Gender (male/female)	106/31	33/14	0.33
Disease duration ≥ 2 years	65/72	18/29	0.31
BMI (kg/m^2^)			0.13
< 18.5	49 (35.8%)	19 (40.4%)	
18.5–24.9	77 (56.2%)	20 (42.6%)	
≥ 24.9	11 (8.0%)	8 (17.0%)	
Age of onset (A, year)			0.60
A1	13 (9.5%)	2 (4.3%)	
A2	100 (73.0%)	36 (76.6%)	
A3	24 (17.5%)	9 (19.1%)	
Disease location, L			0.96
L1	47 (34.3%)	17 (36.2%)	
L2	8 (5.8%)	3 (6.4%)	
L3	82 (59.9%)	27 (57.4%)	
Upper GI involvement (yes/no)	11/126	4/43	1.00
Perianal disease (P, yes/no)	87/50	37/10	0.07
Disease behavior, B			0.16
B1	70 (51.1%)	31 (66.0%)	
B2	42 (30.7%)	12 (25.5%)	
B3	25 (18.2%)	4 (8.5%)	
Surgery (yes/no)	77/60	25/22	0.74
Alb, median (IQR), g/L	40.8 (36.0, 45.0)	42.1 (37.6, 45.2)	0.34
WBC, 10^9/L			0.23
< 3.97	6 (4.4%)	5 (10.6%)	
3.97–9.15	109 (79.6%)	37 (78.7%)	
> 9.15	22 (16.1%)	5 (10.6%)	
Hb, median (IQR), g/L	131.0 (112.0, 142.0)	124.0 (108.0, 139.0)	0.19
PLT, median (IQR), 10^9/L	275.0 (221.0, 333.0)	304.0 (230.0, 392.0)	0.17
CRP, mg/L			0.33
≤ 8	99 (72.3%)	38 (80.9%)	
> 8	38 (27.7%)	9 (19.1%)	
ESR^a^, mm/L			1.00
≤ 20 or ≤ 15	75 (54.7%)	26 (55.3%)	
> 20 or > 15	62 (45.3%)	21 (44.7%)	

*GI* gastrointestinal, *Alb* albumin, *IQR* interquartile range, *WBC* white blood cell, *Hb* hemoglobin, *PLT* platelet count, *CRP* C-reactive protein, *ESR* erythrocyte sedimentation rate

^a^The threshold value for ESR level is 20 mm/L in female and 15 mm/L in male

### Development of the clinical model, pancreatic radiomics and combined model

Univariate analysis identified disease duration, age of onset (A), disease location (L), upper gastrointestinal involvement, disease behavior (B), white blood cell, CRP, and erythrocyte sedimentation rate as potential risk factors for SLR to IFX. After multivariate analysis, disease duration, A, L, and B were chosen as independent predictive factors in the clinical model, as demonstrated in Table 2.

**Table 2:** Univariate and multivariable analysis for SLR to IFX in the training datasets

Characteristics	Univariate analysis			Multivariable analysis		
	OR	95% CI	*p*	OR	95% CI	*p*
Gender	0.621	0.276–1.400	0.25			
Disease duration ≥ 2 years	3.254	1.608–6.762	0.001	3.244	1.425–7.383	0.01
BMI, kg/m^2^						
< 18.5	1.000					
18.5–24.9	0.762	0.366–1.589	0.47			
≥ 24.9	1.111	0.286–4.179	0.88			
Age of onset (A, year)						
A1	1.000			1.000		
A2	2.222	0.634–10.366	0.25	3.508	0.671–18.333	0.14
A3	2.821	0.664–15.008	0.18	7.121	1.035–49.007	0.046
Disease location, L						
L1	1.000			1.000		
L2	18.308	2.873–360.211	0.01	35.751	3.337–382.986	0.003
L3	1.853	0.866–1.290	0.12	2.671	0.996–7.165	0.051
Upper GI involvement	0.316	0.047–1.290	0.15			
Perianal disease, P	1.098	0.540–2.263	0.80			
Disease behavior, B						
B1	1.000			1.000		
B2	1.390	0.626–3.075	0.42	1.494	0.571–3.910	0.41
B3	2.601	1.029–6.751	0.04	3.404	1.123–10.311	0.03
Surgery	0.660	0.329–1.317	0.24			
Alb, g/L	0.970	0.920–1.021	0.25			
WBC, 10^9/L						
< 3.97	1.000			1.000		
3.97–9.15	3.015	0.465–58.836	0.32	2.182	0.224–21.231	0.50
> 9.15	6.000	0.793–125.285	0.13	7.030	0.603–81.971	0.12
Hb, g/L	0.989	0.972–1.006	0.20			
PLT, × 10^9/L	1.002	0.998–1.006	0.23			
CRP, mg/L						
≤ 8	1.000			1.000		
> 8	1.829	0.856–3.924	0.12	2.095	0.821–5.341	0.12
ESR^a^ mm/L						
≤ 20 or ≤ 15	1.000					
> 20 or > 15	1.758	0.882–3.536	0.11			

*GI* gastrointestinal, *Alb* albumin, *WBC* white blood cell, *Hb* hemoglobin, *PLT* platelet count, *CRP* C-reactive protein, *ESR* erythrocyte sedimentation rate

^a^The threshold value for ESR level is 20 mm/L in female and 15 mm/L in male

**Fig. 2:**
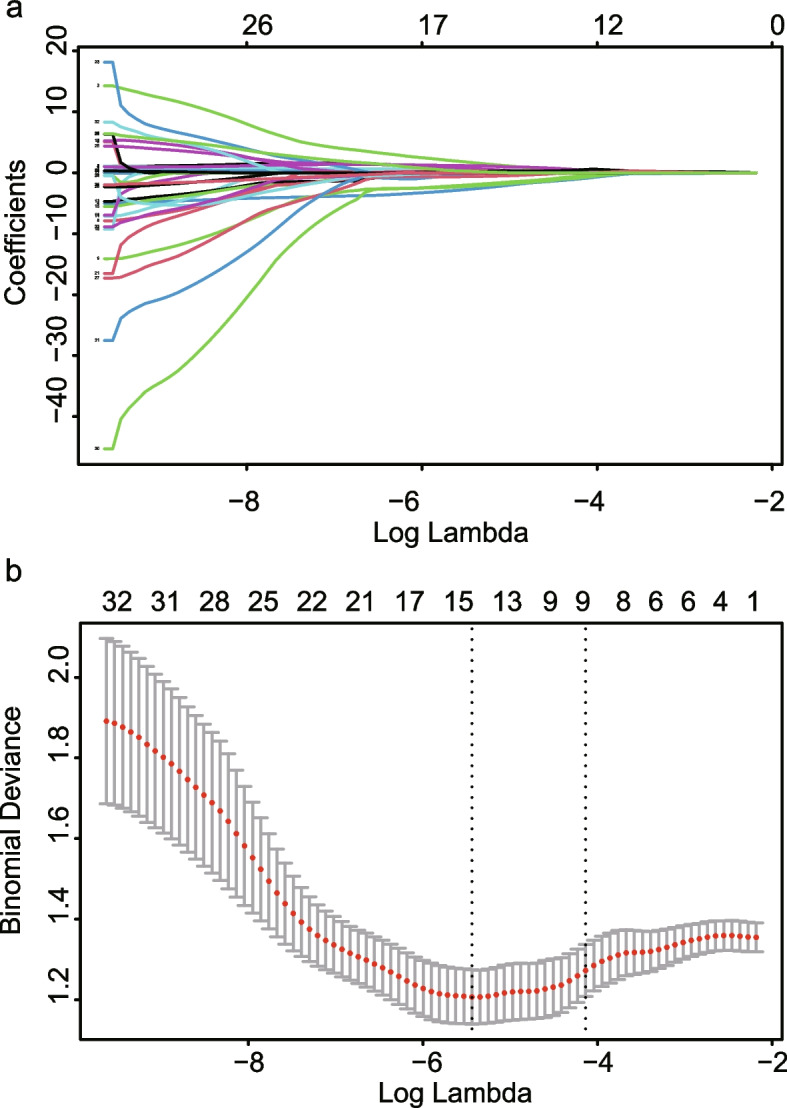
Radiological texture features selection utilizing the least absolute shrinkage and selection operator (LASSO) algorithm and 10-fold cross-validation. **a** Optimal parameter (*λ*) selection in LASSO model used cross-validation via minimum criteria. Dotted vertical lines were drawn at the optimal values by using the minimum criteria and 1 standard error of the minimum criteria (the 1-SE criteria). A *λ* of 0.016 with log (*λ*) =  -4.135 was chosen. **b** LASSO coefficient profiles of the 37 radiomics features with ICC > 0.8. A coefficient profile plot was generated versus the selected log(*λ*) value, where 10 features with nonzero coefficients were chosen

After applying LASSO selection, the pancreatic features with ICC over 0.8 were reduced to ten variables (Fig. 2), which were further simplified into five to construct the pancreatic radiomics model by backward stepwise multivariate logistic regression.

For integrating clinical parameters and radiomics features, the rad_score was formulated by the equation: rad_score =  -0.803 + (-3.061)*CONVENTIONAL_Humax + 2.181*CONVENTIONAL_Hustd + (-1.615)*HISTO_Energy + 1.624*GLRLM_GLNU + (-3.120)*GLZLM_LGZE. Finally, the combined model was built by logistic analysis for differentiating distinct treatment responsiveness (Table 3).

**Table 3:** Multivariate regression analyses of the combined model

Intercept and variables	*β*	OR (95% CI)	*p* value
Intercept	-2.651		0.01
Disease duration	1.303	3.682 (1.377, 9.843)	0.01
A2	0.280	1.324 (0.211, 8.303)	0.76
A3	1.482	4.401 (0.515, 37.595)	0.18
L2	4.740	114.442 (4.441–2949.029)	0.004
L3	1.515	4.549 (1.372, 15.082)	0.01
B2	0.550	1.733 (0.556, 5.404)	0.34
B3	1.484	4.410 (1.128, 17.238)	0.03
Rad_score	1.108	3.028 (1.943, 4.719)	< 0.001

*OR* odds ratio, *CI* confidence interval, *A* age of onset, *L* disease location, *B* disease behavior, *Rad_score* radiomics score

### Model comparison and nomogram building

**Fig. 3:**
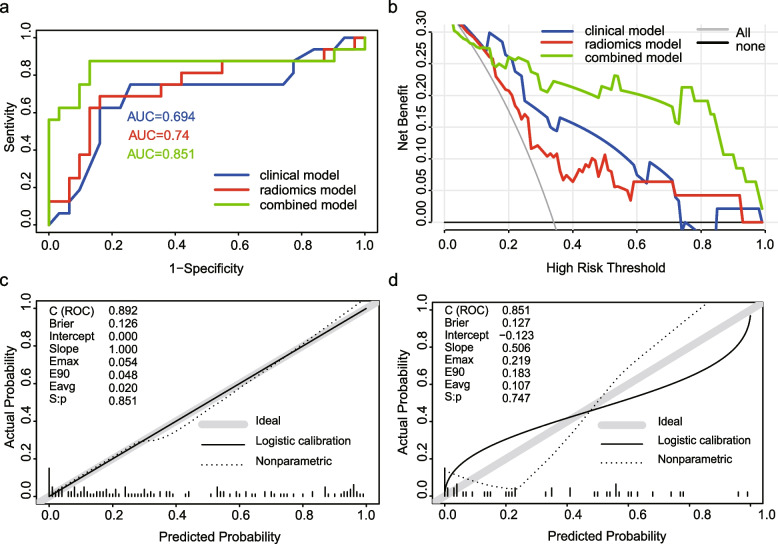
**a** ROC curves for three models in the validation datasets. **b** DCAs for three models in the validation datasets. **c** Calibration plot for combined model in the training cohorts. **d** Calibration plot for combined model in the validation cohorts

**Fig. 4:**
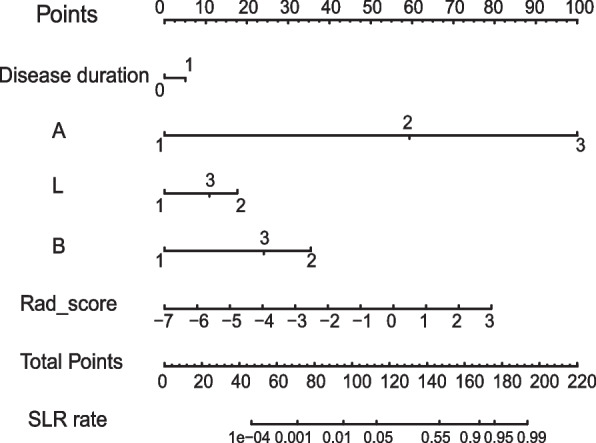
The developed nomogram for predicting secondary loss of response to infliximab in patients with Crohn’s disease

**Fig. 5:**
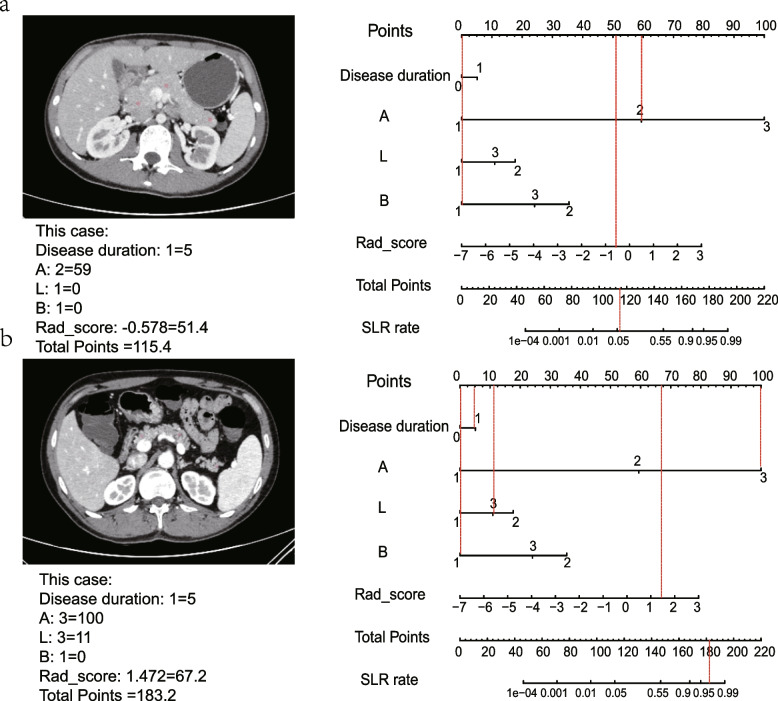
**a** Example of applying nomogram in the patient who benefit from IFX. **b** Example of applying nomogram in the patient who suffer from SLR to IFX

Model comparisons were conducted in the validation cohorts. The combined model showed the lowest AIC value compared with the clinical model and pancreatic radiomics individually (AIC: 127.69 vs 169.56 vs 151.31) and was defined as the optimal model. It also demonstrated satisfactory discrimination for differentiating SLR from non-SLR, with an AUC of 0.851 (95% confidence interval [CI] 0.692–1.000), compared with the clinical model (AUC = 0.694, 95% CI 0.517–0.870) and radiomics model (AUC = 0.740, 95% CI 0.573–0.907) (Table 4). The DeLong test implied that the combined one was statistically excellent than the clinical model (*p* = 0.02) and pancreatic radiomics (*p* = 0.04). In addition, IDI analysis also demonstrated significant improvement in discrimination efficiency of combined models relative to the other models (IDI _clinical model_: 0.145, *p* < 0.001; IDI _radiomics model_ 0.357, *p* < 0.001). The ROC plots and DCA curves of three models were presented in Fig. 3a and b, which suggested that the combined one excelled other model and provided more clinical net benefit in 20–100% risk thresholds. Additionally, the combined model illustrated great consistency between the prediction findings and the actual probabilities in both datasets, as demonstrated in Fig. 3c and d. Founded on the combined model, we constructed a visualized nomogram for simplified application in clinical practice (Figs. 4 and 5). Furthermore, 10-fold cross-validation was conducted to substantiate the robustness of the nomogram, with average AUC, specificity, sensitivity, and accuracy values of 0.872, 0.862, 0.867, and 0.860, respectively (Additional file 2, supplementary material).

**Table 4:** Accuracy and predictive value among three models

	AUC	95% CI	Sensitivity	Specificity	Accuracy
Training cohort					
Clinical model	0.758	0.678–0.839	68.5%	74.7%	72.3%
Radiomics model	0.817	0.745–0.888	85.2%	68.7%	75.2%
Combined model	0.892	0.837–0.947	70.4%	94.0%	84.7%
Validation cohort					
Clinical model	0.694	0.517–0.870	75.0%	74.2%	74.5%
Radiomics model	0.740	0.573–0.907	68.8%	83.9%	78.7%
Combined model	0.851	0.692–1.000	87.5%	87.1%	87.2%

*AUC* area under the curve, *CI* confidence interval

## Discussion

Pancreatic involvement in patients with CD has been suggested to be tightly related to disease activity and non-responsiveness to anti-TNF drugs. Increasing evidence has suggested that TA can be used to assess the risk of treatment failure. Therefore, we established and compared prediction models using clinical baseline characteristics and pancreatic CTE texture features. The combined model was found to be superior to other models, as corroborated by the ROC analysis, IDI, calibration power, and clinical usage. Moreover, as far as we are aware, this is the first attempt to establish a nomogram integrating pancreatic texture features and clinical features to assess the possibility of treatment failure in CD individuals receiving IFX therapy in order to help clinicians offer personalized decision-making.

Using logistic regression analyses, four clinical candidate variables were considered to predict SLR. In our study, individuals with older age at disease diagnosis were found to have poor treatment outcomes, which was not surprising as some studies have substantiated the linkage between age at IFX onset and long-term outcomes [40, 41]. Of particular note, Vermeire et al. demonstrated that older age at disease initiation could be utilized to predict primary loss of response to IFX therapy in CD patients [42]. Researchers speculated that late-onset IBD patients exhibited adverse responsiveness to IFX possibly due to age-related immune system changes caused by alterations in immune cells and gut flora composition, leading to different disease pathogenesis [43]. However, these speculative mechanisms require further verification. Our study also identified that individuals with isolated ileitis disease (Montreal Classification L1) were more likely to benefit from IFX treatment compared to those with colonic involvement. Similarly, a meta-analysis demonstrated that patients with colon involvement had higher loss of response incidence that those without [44]. In concordance, some studies substantiated that patients with isolated colonic disease (Montreal Classification L2) were at a higher risk of treatment failure, possibly due to a larger proportion of fecal IFX loss [44, 45]. In addition, as with other research, our study showed that patients with non-stricturing and non-penetrating diseases (Montreal Classification B1) tended to have a more beneficial response to anti-TNF agents than stenosing disease (B2) or fistulizing disease (B3) [8, 46, 47]. Especially, patients with fibrostenotic phenotype might be more appropriate to receive endoscopic or surgical treatment [48]. In addition, individuals with a disease duration longer than 48 months had diminished responsiveness to anti-TNF drugs compared to those with a shorter disease course. This is consistent with the findings of other groups [49, 50], which could be attributed to several factors, such as altered mucosal cytokine profiles [51] and advanced fibrosing organ damage [48].

Compared with previous studies that focused on identifying clinical factors related to SLR to IFX, our research not only explored clinical characteristics, but also attempted to mine CTE texture features. Chen et al. demonstrated that CTE texture features could be used to predict SLR in patients with CD administered IFX [26]. Taking the underlying associations between pancreatic involvements and treatment failure, five radiomics features were selected, including CONVENTIONAL_Humax, CONVENTIONAL_Hustd, HISTO_Energy, GLRLM_GLNU, and GLZLM_LGZE. CONVENTIONAL indices measure the mean, minimum, maximum, and standard deviation value in the ROIs. Higher CONVENTIONAL_Hustd and Lower CONVENTIONAL_Humax confer risk for adverse response to anti-TNF agents, which may be due to high lesion heterogeneity and more extensive and severe pancreatic destruction, separately. In addition, HISTO_Energy, GLRLM_GLNU, and GLZLM_LGZE respectively reflect the uniformity of the distribution, the similarity of image grayscale values, and the distribution of the low grey-level zones [52]. In this study, GLRLM_GLNU were higher, HISTO_Energy and GLZLM_LGZE were lower in non-responders than in responders, which may indicate that the higher heterogeneity and impaired homogeneity in CTE image, the higher risk of loss of response (Additional file 3, supplementary material). Loss of heterogeneity and improving homogeneity on the treatment/follow-up CT are known indicators of neoadjuvant chemotherapy response in primary esophageal cancer [53]. Then we constructed a pancreatic radiomics model and combined model incorporating clinical and radiomics feature, and compared these models with clinical model to explore the utility of pancreatic TA in predicting long-term treatment outcomes of IFX. We found that the combined model was better in terms of AUC analysis (0.851 vs. 0.694), IDI analysis, calibration ability, and clinical practical value. As a result, we propose a combined nomogram to extrapolate to the clinic for assisting clinicians in making personalized decisions, which illustrated that the rad_score plays an indispensable part in assessing therapy effectiveness, highlighting the significance of pancreatic texture analysis.

Certain limitations of our study should be acknowledged. The single-center retrospective study design limited the clinical practicality of the nomogram and qualitative assessment of pancreatic condition (EIMs or complication). Further multicenter prospective external validation is required to avoid selection bias, verify the generalizability of the nomogram, and clarify the causes of pancreatic manifestations in patients. However, it is worth noting that this study aims to explore the relationship between pancreatic manifestations and IFX efficacy and does not limit pancreatic manifestations to EIMs or complications or other factors. Of course, further clarification of the causes of pancreatic conditions is beneficial to conduct in-depth research and refine conclusions. In addition, owing to the finite sample size, our conclusions may not be sufficiently convincing and require further optimization and verification in larger cohorts. Meanwhile, a larger queue is conducive to further explore the correlation between pancreatic clinical features and IFX responsiveness. In view of this, a post hoc analysis of sample size is performed, which finds that although the current sample size does not fully meet the criteria proposed by Riley et al. (*n* = 289; events per predictor parameter [EPP]: 14.45) [54], the EPP of the current model reaches 12.512. Furthermore, instability plot based on bootstrap model (*b* = 500 times) demonstrates that the existing model is relatively stable (mean absolute percentage error: 0.0585) [55, 56]. Additionally, the definition of the outcome in this study is not sufficiently objective. It is well established that endoscopic healing is regarded as the gold standard in clinical practice [57]. However, most individuals do not routinely undergo endoscopy at 54 weeks of IFX treatment. Thus, studies evaluating treatment outcomes through endoscopy should be conducted in the future.

In conclusions, this study has provided evidence that the clinical variables and pancreatic radiomics features extracted from CTE images can be utilized as non-invasive markers for SLR risk prediction in individuals with CD. The nomogram demonstrated impressive predictive ability with great discriminatory power and obvious clinical benefits. Thus, it could aid in selecting the most appropriate biological scheme for individual patients. Finally, we also indicated that coexisting conditions in CD patients might play a role in predicting responsiveness to biologic agents and ought to be taken into consideration when customizing treatment.

### Supplementary Information

**Additional file 1.** Features extracted from pancreatic areas in CT enterography images.

**Additional file 2.** AUC and accuracy of the combined model in internal validation.

**Additional file 3.** Multivariate Regression Analyses of the Radiomics Model.

## Data Availability

The data underlying this article available from the corresponding author on reasonable request.

## Abbreviations

A: Age of onset
AIC: Akaike information criterion
AIP: Autoimmune pancreatitis
Anti-TNF: Anti-tumor necrosis factor
AUC: The area under the ROC curve
B: Disease behavior
CD: Crohn’s disease
CI: Confidence interval
CRP: C-reactive protein
CTE: Computed tomography enterography
DCA: Decision curve analysis
EIMs: Extraintestinal manifestations
EPP: Events per predictor parameter
HBI: Harvey Bradshaw indices
IBD: Inflammatory bowel disease
ICC: Intraclass correlation coefficient
IDI: Integrated discrimination improvement
IFX: Infliximab
L: Disease location
LASSO: Least absolute shrinkage and selection operator
Rad_score: Radiomics score
ROC: Receiver operating characteristic
ROI: Regions of interest
SES-CD: Simple endoscopic score for CD
SLR: Secondary loss of response
TA: Texture analysis
