# Supplementary material for: CT-based pancreatic radiomics predicts secondary loss of response to infliximab in biologically naïve patients with Crohn’s disease
Source: Insights Imaging. 2024 Mar 13;15:69. doi: 10.1186/s13244-024-01637-4 (PMC10933237; doi:10.1186/s13244-024-01637-4)
Supplement: Supplementary file 1 — Additional file 1. Features extracted from pancreatic areas in CT enterography images. [file 13244_2024_1637_MOESM1_ESM.pdf]

CT-based pancreatic radiomics predicts secondary loss of response to infliximab in biologically naïve patients with Crohn's disease

ELECTRONIC SUPPLEMENTARY MATERIAL

Additional file 1: Features extracted from pancreatic areas in CT enterography images.

Matrix	Index
Conventional features	CONVENTIONAL_HUmin<br>CONVENTIONAL_HUmean<br>CONVENTIONAL_HUstd<br>CONVENTIONAL_HUmax
Histogram features	HISTO_Skewness<br>HISTO_Kurtosis<br>HISTO_ExcessKurtosis<br>HISTO_Entropy_log10<br>HISTO_Entropy_log2<br>HISTO_Energy
Shape features	SHAPE_Volume (mL)<br>SHAPE_Volume (# vx)
Grey-level co-occurrence matrix (GLCM)	GLCM_Homogeneity<br>GLCM_Energy<br>GLCM_Contrast<br>GLCM_Correlation<br>GLCM_Entropy_log10<br>GLCM_Entropy_log2<br>GLCM_Dissimilarity
Grey-level run-length matrix (GLRLM)	GLRLM_SRE (Short-Run Emphasis)<br>GLRLM_LRE (Long-Run Emphasis)<br>GLRLM_LGRE (Low Gray-level Run Emphasis)<br>GLRLM_HGRE (High Gray-level Run Emphasis)<br>GLRLM_SRLGE (Short-Run Low Gray-level Emphasis)<br>GLRLM_SRHGE (Short-Run High Gray-level Emphasis)<br>GLRLM_LRLGE (Long-Run Low Gray-level Emphasis)<br>GLRLM_LRHGE (Long-Run High Gray-level Emphasis)<br>GLRLM_GLNU (Gray-Level Non-Uniformity for run)<br>GLRLM_RLNU (Run Length Non-Uniformity)<br>GLRLM_RP (Run Percentage)
Neighbourhood grey-level different matrix (NGLDM)	NGLDM_Coarseness<br>NGLDM_Contrast<br>NGLDM_Busyness
	GLZLM_SZE (Short-Zone Emphasis)<br>GLZLM_LZE (Long-Zone Emphasis)<br>GLZLM_LGZE (Low Gray-level Zone Emphasis)

Grey-level zone-length matrix (GLZLM)	GLZLM_HGZE (High Gray-level Zone Emphasis)<br>GLZLM_SZLGE (Short-Zone Low Gray-level Emphasis)<br>GLZLM_SZHGE (Short-Zone High Gray-level Emphasis)<br>GLZLM_LZLGE (Long-Zone Low Gray-level Emphasis)<br>GLZLM_LZHGE (Long-Zone High Gray-level Emphasis)<br>GLZLM_GLNU (Gray-Level Non-Uniformity for zone)<br>GLZLM_ZLNU (Zone Length Non-Uniformity)<br>GLZLM_ZP (Zone Percentage)
