# Supplementary material for: CT-based pancreatic radiomics predicts secondary loss of response to infliximab in biologically naïve patients with Crohn’s disease
Source: Insights Imaging. 2024 Mar 13;15:69. doi: 10.1186/s13244-024-01637-4 (PMC10933237; doi:10.1186/s13244-024-01637-4)
Supplement: Supplementary file 2 — Additional file 2. AUC and accuracy of the combined model in internal validation. [file 13244_2024_1637_MOESM2_ESM.pdf]

Additional file 2: AUC and accuracy of the combined model in internal validation

Fold	AUC	Sentitivity	Specificity	Accuracy
1	1.000	1.000	1.000	1.000
2	0.989	1.000	0.909	0.947
3	0.821	0.714	1.000	0.895
4	0.917	1.000	0.833	0.889
5	0.870	0.857	0.818	0.833
6	0.769	0.833	0.769	0.789
7	0.875	0.833	0.917	0.889
8	0.935	0.727	1.000	0.833
9	0.875	0.833	0.917	0.889
10	0.670	0.875	0.455	0.632
Mean	0.872	0.867	0.862	0.860

AUC, area under the curve.
