# Supplementary material for: CT-based pancreatic radiomics predicts secondary loss of response to infliximab in biologically naïve patients with Crohn’s disease
Source: Insights Imaging. 2024 Mar 13;15:69. doi: 10.1186/s13244-024-01637-4 (PMC10933237; doi:10.1186/s13244-024-01637-4)
Supplement: Supplementary file 3 — Additional file 3. Multivariate Regression Analyses of the Radiomics Model. [file 13244_2024_1637_MOESM3_ESM.pdf]

Additional file 3: Multivariate Regression Analyses of the Radiomics Model

Intercept and Variables	β	OR （95% CI）	P value
Intercept	-0.803		
CONVENTIONAL_Humax	-3.061	0.047(0.011-0.193)	<0.001
CONVENTIONAL_Hustd	2.181	8.854(2.677-29.287)	<0.001
HISTO_Energy	-1.615	0.199(0.076-0.520)	<0.001
GLRLM_GLNU	1.624	5.072(2.171-11.854)	<0.001
GLZLM_LGZE	-3.120	0.044(0.010-0.190)	<0.001

OR, odds ratio; CI, confidence interval.
